# Thin Wall Milling at a Maximized Axial Depth of Cut

**DOI:** 10.3390/ma18225219

**Published:** 2025-11-18

**Authors:** Magdalena Zawada-Michałowska

**Affiliations:** Department of Production Engineering, Lublin University of Technology, Nadbystrzycka 38D, 20-618 Lublin, Poland; m.michalowska@pollub.pl

**Keywords:** milling, thin-walled element, aluminum alloy, residual stress, deformation, ANOVA, RSM, DoE

## Abstract

The objective of the study was to determine the minimum thickness of a thin wall for milling at a maximized axial depth of cut, considering the effect of cutting speed on residual stress and post-machining distortion. Test samples were made of aluminum alloy 7050 T7451. The milling operation at a maximized axial depth of cut was performed during finishing. Response surface methodology was employed. Wall thickness and cutting speed were considered as two independent variables, while dependent variables were flatness deviation, wall thickness deviation, and residual stress. Flatness deviation and wall thickness deviation were used as the indicators of post-machining wall deformation and their measurements were made using a coordinate measuring machine. Residual stress was measured with an X-ray diffractometer. The obtained results showed that thin wall milling at a maximized axial depth of cut was feasible; nevertheless, for a wall thickness of *t* = 1 mm, the formation of considerable post-machining deformation was observed. Therefore, for milling with the employed axial depth of cut, the wall thickness should be *t* ≥ 1.5 mm. The highest strain and residual stress were observed at *v_c_* ≈ 600 m/min; despite its subsequent decrease, the strain at *v_c_* = 900 m/min was still higher than that at *v_c_* = 300 m/min. The results also showed tensile stress to be dominant, while compressive stress only occurred at *v_c_* = 300 m/min for wall thicknesses of *t* = 1.5 mm and *t* = 2 mm. The developed response surface quadratic models make it possible to predict the tested variables under similar conditions.

## 1. Introduction

Advancements in the aviation industry are largely based on materials and structural innovations that are designed to enhance aircraft operating performance [1,2]. One of the key trends is to use integral thin-walled components that enable reduced weight and, at the same time, high strength of such structures [3]. Although the literature of the subject offers a vast number of definitions of a thin-walled element, it is generally assumed that such part consists of a thin plate and stiffening, with its wall thickness lower than 2.5 mm [4]. The application of thin-walled elements allows for improved aerodynamical parameters along with reduced fuel consumption and gas emissions, which is particularly important in terms of more and more stringent environmental and economic requirements [5,6]. The manufacture of these components for aircraft poses numerous technological challenges. Given the fact that such components are low-rigidity structures [7,8], they are particularly prone to deformation during machining [9]. As a result, their dimensional and shape accuracy is difficult to achieve [10]. It implies significant financial losses generated by the rejection of defective pieces [11]. In light of the above, studies devoted to optimizing and improving the effectiveness of machining such parts constitute a key research area [12].

In industrial practice, milling is a widely used method for producing integral thin-walled elements [13]. Semi-finished products are usually fabricated from monolithic rolled plates, nearly 90% of which are often converted into chips [14,15]. These components are usually made of light-metal alloys, with aluminum alloys being predominant in this group [16,17]. The relatively low density of the materials contributes to their widespread use in structural applications [18,19,20]. The formation of post-machining distortions in thin-walled parts is affected by numerous factors, which can generally be classified as factors relating to the geometry and material of a workpiece (product dimensions, thin wall thickness, physical properties, etc.) and factors relating to the machining process itself (technological parameters, tool type and its path, cooling method, etc.) [21]. Li et al. [22] divided post-machining deformation into two categories: mechanically and thermally induced deformation, stressing that they usually stem from a complex interaction of various parameters. One of the major sources of post-machining distortions in thin-walled components is the occurrence of residual stress [23,24,25], a phenomenon that is an important characteristic of surface integrity [26,27]. According to Chighizola et al. [28] and Huang et al. [29], residual stress can be classified into: initial residual stress (bulk residual stress) and machining-induced residual stress. Initial residual stress is an effect of the prior processing (i.e., technological history) of a semi-finished product, while machining-induced residual stress occurs during machining. Akhtar et al. [30] examined the combined influence of initial residual stress and machining-induced residual stress, demonstrating that the initial residual stress primarily affected workpiece deformation in roughing, whereas the machining-induced residual stress was key during finishing. In addition, for wall thicknesses lower than 4–5 mm, machining-induced residual stress was identified as the main contributor. Masoudi et al. [31] highlighted the difference in the location of the these stress types, showing that initial residual stress was distributed across the entire volume of the material, in contrast, machining-induced residual stress was confined to a thin layer generated by the impact of the cutting tool on the workpiece. Cerutti and Mocellin [32] stressed that the use of stress-relieving processes following semi-finished part production would seldom result in a total removal of residual stress. Initial residual stress is partly “released” during machining, while at the same time, machining-induced residual stress is produced. This disturbs the initial equilibrium, leading to the formation of a new stress state, which finally causes part distortion. The mechanism of generating machining-induced residual stress is intricate. It results from thermal and mechanical interactions in the cutting zone [33,34]. An analysis of residual stress-inducing mechanisms is of vital importance in terms of machining process optimization, because it allows a better understanding of the relationships between cutting parameters and surface layer properties [35,36].

As thin-walled parts are prone to deflection and hence their machining poses numerous challenges related to accuracy and quality [37], many researchers studied the issue of post-machining deformation, proposing different solutions for reducing this problem by optimizing either machining conditions or machining strategy, including tool path [38]. Table 1 offers a compilation of relevant studies on minimizing the post-machining deformation of thin-walled elements made of aluminum and titanium alloys.

**Figure 1:**
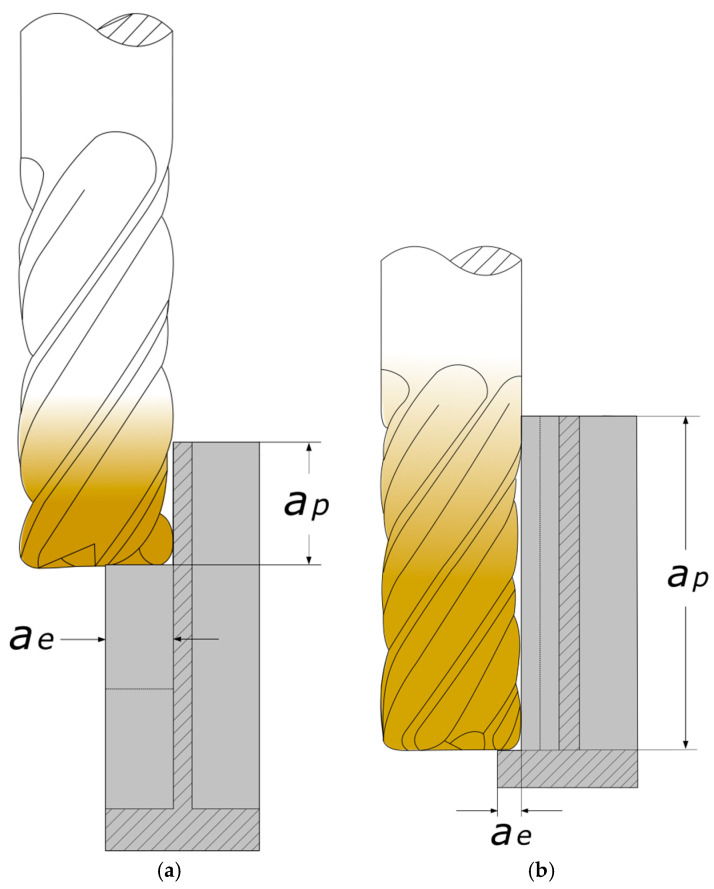
Thin wall milling: (**a**) standard approach; (**b**) milling at a maximized axial depth of cut: *a_p_*—axial depth of cut, *a_e_*—radial depth of cut (milling width).

In effect, many new technological concepts have been developed to improve product quality. The conventional approach is to use machining strategies for thin-walled part milling, such as the “Christmas tree” strategy (it is recommended when the wall height-to-thickness ratio is greater than 30:1) and alternate milling (it is recommended when the wall height-to-thickness ratio is less than 30:1) [50,51]. Recently, cutting tool manufacturers have presented the benefits of thin wall machining conducted over the entire height of the workpiece [52,53,54], which is called milling at a maximized axial depth of cut (Figure 1). It consists of engaging the cutting edge over the full height of the thin wall. This allows for reducing the number of tool passes (in standard milling conducted with a small axial depth of cut, many passes have to be performed to achieve the required wall height [55,56]) and a more efficient use of the cutting tool, leading to a considerable reduction in technological cycle time and production costs, as well as increased productivity of the machine tool. An important aspect is also the absence of machining marks at individual levels corresponding to the axial depth of cut, which also affects the quality of the manufactured part. Theoretically (in compliance with the information given by manufacturers of such tools), this should also reduce the risk of post-machining distortions in thin-walled parts. However, the solution requires the application of cutting tools with specially designed geometry characterized by diversified pitch and variable helix angle. It should be emphasized that there is no universal standard for thin wall milling at maximized axial depths of cut. In industrial practice, the selection of appropriate parameters is predominantly determined through trial-and-error and relies greatly on the experience and expertise of process engineers supervising machining processes.

Milling at a maximized axial depth of cut is characterized by the occurrence of springback, a phenomenon involving elastic deformation of a thin wall during machining. As a result of the forces exerted by the tool, the upper part of the wall is deflected and only partially returns to its original shape after the process. Consequently, the cross-section of the wall often assumes the profile of a trapezoid, with the wall thickness being the highest at the top and the smallest at the base [57,58]. It should be emphasized that the machining of thin, especially high, walls requires the use of tools with a considerable working part length and thus a significant tool overhang length. In effect, both the workpiece and the tool are deformed by the cutting force [59], which has an impact on product quality and process effectiveness [60].

Despite numerous studies on thin wall machining, milling at a maximized axial depth of cut has not been adequately investigated. Most research works focus on minimizing deflection through reduced axial depth of cut or optimized tool paths, leaving a gap in understanding how this cutting solution affects residual stress formation and post-machining deformation. Thin wall milling at a maximized axial depth of cut poses a considerable technological challenge due to the necessity of ensuring high effectiveness of the process and maintaining the required dimensional and shape accuracy of these parts. Therefore, this study examines the influence of wall thickness and cutting speed on the machining quality of thin wall milled at a maximized axial depth of cut. Based on the authors’ practical experience, wall thickness and cutting speed (besides the radial depth of cut, i.e., milling width) are the key parameters governing manufacturing accuracy under such conditions.

The objective of the study is to establish the minimum wall thickness suitable for milling at a maximum axial depth of cut and, at the same time, to determine the effect of cutting speed on residual stress and post-machining deformation of such a wall. The obtained results can serve as a basis for developing technological guidelines for industry.

## 2. Materials and Methods

**Figure 2:**
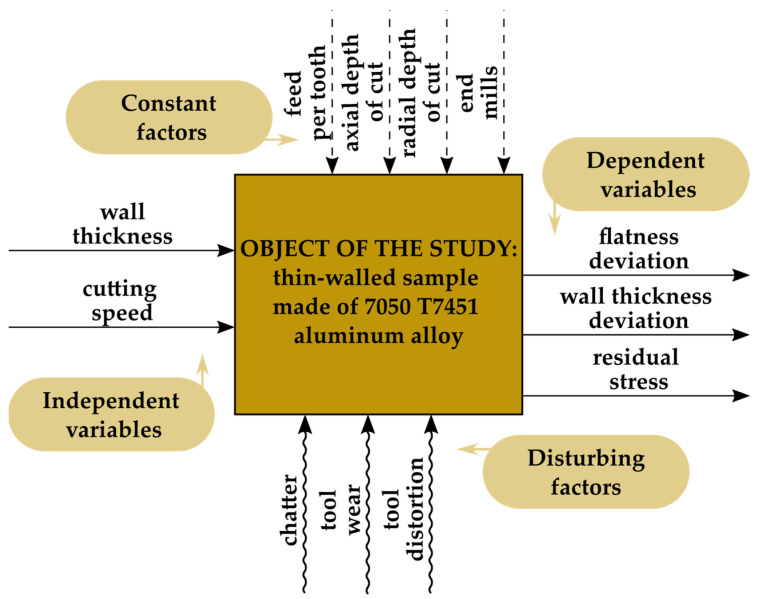
Research plan.

Figure 2 shows the adopted research plan. The study object was a thin-walled sample (with variable wall thickness) made of aluminum alloy 7050 T7451. Independent variables were wall thickness and cutting speed. Dependent variables included flatness deviation, wall thickness deviation, and residual stress. Constant factors were feed per tooth, axial depth of cut, radial depth of cut (milling width), and end mills. Disturbing factors included chatter and tool wear, as well as tool deflection.

The study was performed in accordance with the design of experiment (DoE) approach, which helps determine independent variables having a significant effect on a process in question, develop a mathematical model fitted to experimental data, and optimize the process by finding the best parameter setting [61]. The Design-Expert^®^ (23.1.8) software was employed for both the experimental design and results analysis. Response surface methodology (RSM) [62] and face-centred central composite design (FCCCD) were employed. The optimized number of experimental trials was set to 13. In compliance with the experimental design, the central point was repeated 5 times. Experimental data were analyzed using coded variables. The actual values of the independent variables were converted into dimensionless coded values using the normalization approach shown in Equation (1):

(1) $$x_{i}=\frac{X_{i}-X_{i,0}}{\Delta X_{i}}$$

where *x_i_*—the coded value of the *i*-th independent variable, *X_i_*—the actual value of the *i*-th independent variable, *X_i_*_,0_—the actual value of the *i*-th independent variable at the centre point, Δ*X_i_*—the step change value of *X_i_* which was calculated according to Equation (2):

(2) $$\Delta X_{i}=\frac{X_{i,max}-X_{i,min}}{2}$$

where *X_i_*_,*max*_—the maximum actual value of the *i*-th independent variable, *X_i_*_,*min*_—the minimum actual value of the *i*-th independent variable.

In Table 2 are listed independent variables and their coded levels for the face-centred central composite design. Wall thickness and cutting speed, i.e., independent variables, are denoted by *A* and *B*. Levels of each variable are represented by coded forms as follows: −1 (low), 0 (medium), and +1 (high).

**Figure 3:**
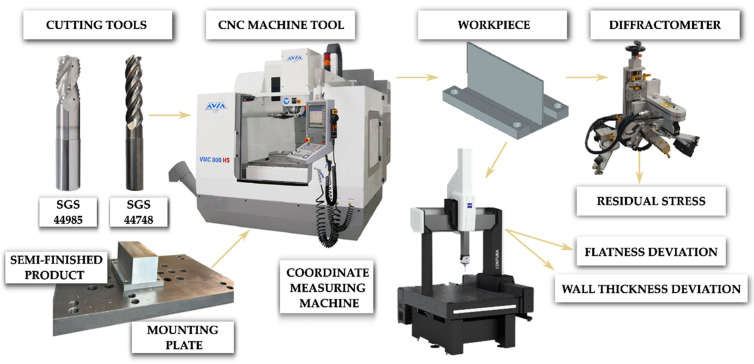
Experimental setup.

Figure 3 shows the employed experimental setup, including a CNC machine tool, cutting tools, semi-finished product and its mounting, workpiece after machining, as well as measuring equipment (diffractometer and coordinate measuring machine).

Thin-walled samples (with variable wall thickness) were made of aluminum alloy 7050 T7451, compliant with the AMS4050K standard [63], exhibiting high mechanical properties (tensile strength *R_m_* = 520 MPa, yield strength *R_p_*_0.2_ = 450 MPa, elongation *A*_5_ = 13%) and good stress corrosion resistance. This material is commonly used in aerospace (fuselage frames, wing skins, bulkheads, and other structural components) and military applications (high-performance structural components and transport equipment). Table 3 gives its chemical composition.

The rectangular test samples had a length of 100 mm and a width of 65 mm. They were fabricated from a 50 mm thick rolled plate. Their pretreatment consisted of preparation for mounting the workpiece in a specially designed mounting plate by means of screw joints for repeatability. Following the proper treatment, the tested thin wall had a height of 48 mm (it was axial depth of cut), which resulted from the base thickness equal to 2 mm. As previously mentioned, the wall thickness was an independent variable, and it was set to 1, 1.5, and 2 mm.

Milling was carried out on a CNC machining centre, Avia VMC 800HS (Poland, Warsaw). The proper down-cutting process was conducted dry in two operations: roughing and finishing. The finishing operation was performed over the entire axial depth of cut, which is the research subject of this study. The focus on this aspect can be justified by the fact that this particular stage of machining has the most significant impact on the quality of finished parts. Technological parameters of roughing and finishing operations are given in Table 4.

Cutting tools from KYOCERA SGS Precision Tools (Cuyahoga Falls, OH, USA) were used. A 44985-type end mill for roughing and a 44748-type end mill for finishing. Both end mills are high-efficiency tools for aluminum alloys. They were mounted in ERICKSON AHPVTTMQL1C heat shrink tool holders 16090M (44985) and 12090M (44748) with an HSK63 tool taper, manufactured by M & J Tooling LLC (Dayton, OH, USA). It must be stressed that the 44748 is recommended for maximum axial depth of cut machining of walls. Detailed technical parameters of the end mills are given in Table 5.

**Figure 4:**
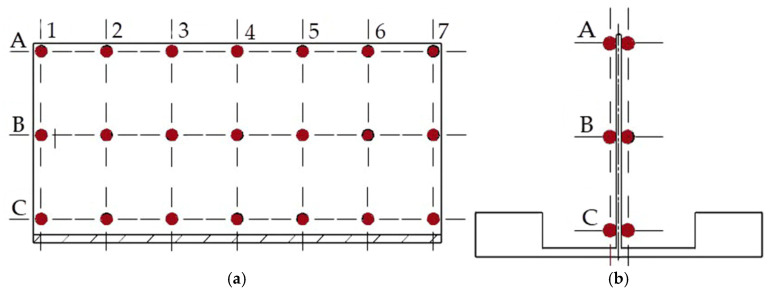
Scheme showing the arrangement of measurement points selected for wall thickness analysis: (**a**) side view; (**b**) front view.

The effects of the finishing treatment, which involved thin wall milling at the maximum axial depth of cut, were described by dependent variables: flatness deviation and wall thickness deviation. The variables were assumed to be the indicators of post-machining deformation of the walls under study. Measurements were made using a coordinate measuring machine, Zeiss Contura 7/10/6 (Oberkochen, Germany), provided with a VAST XT GOLD (Oberkochen, Germany) scanning probe featuring a stylus with a tip diameter of 3 mm, compatible with the Calypso (7.6.04) software. The use of active scanning technology enabled the collection of over 20,000 measurement points. Obtained flatness deviation results allowed for both quantitative and qualitative assessment of wall deformation and 3D visualization of its distribution, thanks to the high sampling density. As for wall thickness results, these measurement points were further processed using points forming a measurement grid (Figure 4) to calculate wall thickness at specified locations. The results showed that the highest deviations were consistently located at the thin wall ends (denoted by 1 and 7), near its top edge (denoted by A). It must be stressed that the CALYPSO measurement report did not reflect the problem of wall trapezoidal distortion; therefore, an additional analysis was performed based on the generated file containing the coordinate values of individual points.

Wall thickness deviation (Δt) was calculated as a difference between the maximum thickness *t_max_* (measured at the top of the wall) and the nominal thickness *t_N_* (3):

(3) $$\Delta t=t_{max}-t_{N}$$

The measurement methodology developed for the coordinate measuring machine provides the basis for investigating correlations between the milling process parameters and the dimensional and shape accuracy of the thin-walled component.

Another dependent variable was residual stress, which was measured with a portable X-ray diffractometer of GNR Theta-Theta EDGE (Agrate Conturbia, Italy) via non-destructive testing. The measurement principle was based on the phenomenon of X-ray diffraction and described by the Bragg Equation (4), stating that diffraction occurs if the product of the integer (n) and the radiation wavelength (λ) equals twice the distance between the crystallographic planes ($2d_{hkl}$) multiplied by the sine of the angle of incidence of the radiation beam on a given crystallographic plane (θ):

(4) $$n\lambda =2d_{hkl}sin\theta$$

**Figure 5:**
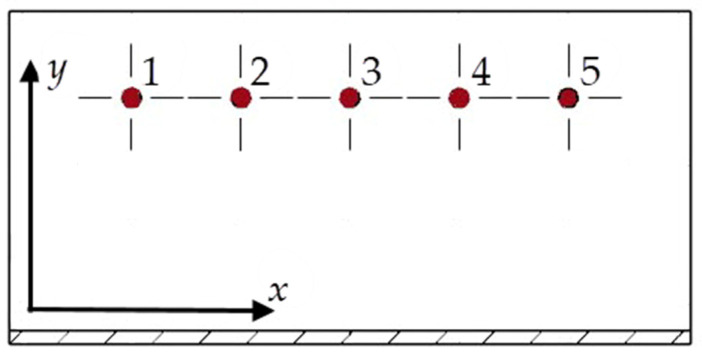
Scheme showing the arrangement of measuring points selected for the analysis of residual stress, together with the designation of the axes.

Residual stress was measured along two axes (*x*, *y*) at the top of the wall at five points, as shown in Figure 5. The experiment was conducted using a 4 W X-ray tube with a chromium anode dedicated to the examination of aluminum alloys. Radiation was focused using a 1 mm diameter collimator, yielding a penetration depth of several micrometres. This enabled a thorough analysis of the residual stress in the surface layer, constituting a key aspect for the functional properties and durability of manufactured components.

Residual stress measurements were carried out using the sin^2^*ψ* measurement method for eleven positions of the *ψ* angle ranging from od –35° to +35°. For each setting, nine signal acquisitions were recorded with the exposure time set to 30 s. To allow the measurement along the *y*-axis, the section of the sample with mounting holes had to be cut off.

The experimental design and results of the face-centred central composite design (FCCCD) are presented in Table A1.

## 3. Results

### 3.1. Flatness Deviation

The first stage of results analysis focused on flatness deviation, one of the indicators of post-machining deformation of thin walls. Both quantitative and qualitative assessments were carried out, with the flatness deviation results also presented in the form of 3D spatial maps to examine the nature of wall deformation. First of all, however, the experimental results were subjected to statistical analysis, and an empirical equation was found for predicting the flatness deviation as a function of the independent variables under study. For this purpose, analysis of variance (ANOVA) was applied, which showed that the best fit was obtained for the quadratic equation, yielding *R*^2^ = 0.9877, adjusted *R*^2^ = 0.9789, and predicted *R*^2^ = 0.8976. The difference below 0.2 between the predicted *R*^2^ and the adjusted *R*^2^ implies reasonably high agreement. The *R*^2^ value approaching 1 indicates a good fit of the model to the experimental data. Also, the adequate precision of 33.6032 confirms a good quality of the signal. The empirical model of flatness deviation (*f_d_*) relative to the independent variables is described by Equation (5) using coded variables:

(5) $$f_{d}=0.4083-0.0557A+0.0298B+0.0027AB+0.0260A^{2}-0.1625B^{2}$$

ANOVA was employed to validate the assumed response surface quadratic model. This model was found to be statistically significant, as evidenced by an *F*-value of 112.15, indicating that the probability of this result arising from noise, i.e., aberrations caused by external influence, is merely 0.01%. An analysis of the *p*-values revealed that most model terms were statistically significant (*p*-value should be less than 0.05). It was found that wall thickness had a significant impact on flatness deviation; the cutting speed was a significant factor, too, yet its impact on flatness deviations was weaker than that of wall thickness. The only exception was observed for the *AB* interaction, which means that the effect of wall thickness and cutting speed is not synergistic, and thus they affect flatness deviation rather independently. Also, the lack of fit *F*-value amounting to 4.90 suggests that the lack of fit is statistically insignificant relative to the pure error, confirming a good fit of the model to the experimental data. The probability that the observed lack of fit *F*-value arises from random noise is 7.93%. In Table 6 are given detailed ANOVA results for the response surface quadratic model of flatness deviation.

**Figure 6:**
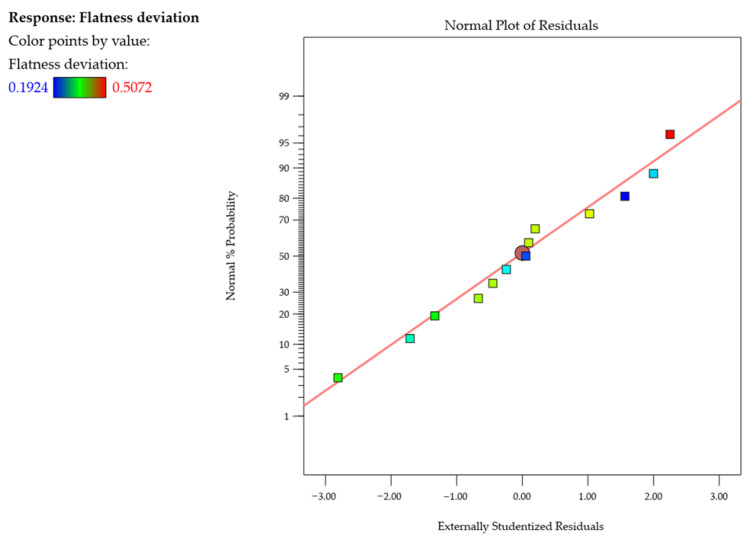
Normal probability plot of studentized residuals for the flatness deviation.

After that, the normal probability plot of studentized residuals was examined (i.e., the difference between an observed and predicted value for estimating the standard deviation, which is used in statistical analysis for outlier identification) for flatness deviation (Figure 6). No response transformation or evident problems with normal distribution were observed. This plot verifies the normality assumption of the model, showing that the residuals follow a straight line. It suggests that the model is adequate.

**Figure 7:**
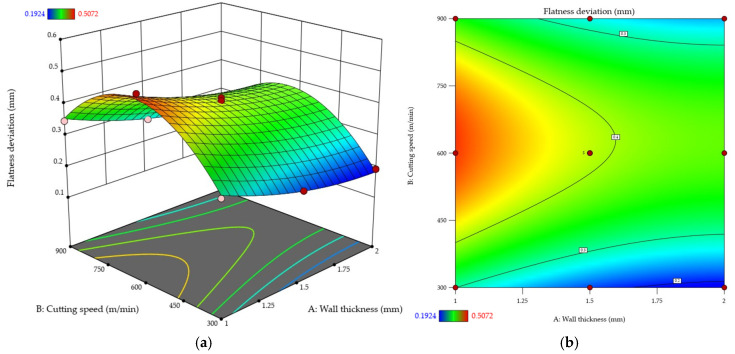
Flatness deviation as a function of wall thickness and cutting speed: (**a**) response surface plot; (**b**) contour plot.

Relationships between the independent variables and the flatness deviation are presented in Figure 7. The analysis was performed based on response surface (Figure 7a) and contour (Figure 7b) plots. The results demonstrate that increasing wall thickness reduces flatness deviation, which confirms that the thicker the wall is, the less deformation-prone this wall becomes. The correlation between cutting speed and flatness deviation is more complex. For each wall thickness tested, the flatness deviation rises as the cutting speed is increased, reaching the maximum at approximately *v_c_* ≈ 600 m/min; although the deviation value begins to decrease afterwards, at a cutting speed of *v_c_* = 900 m/min it is still higher than at *v_c_* = 300 m/min. It should also be emphasized that for a wall thickness of *t* = 1 mm, raising the cutting speed leads to a more rapid escalation of the flatness deviation, while with thicker walls this effect is less powerful. In addition, the difference in the flatness deviation values between wall thicknesses of *t* = 1.5 mm and *t* = 2 mm is not as pronounced as that observed for *t* = 1 mm. It can therefore be concluded that wall thickness strongly affects responsiveness to cutting speed. Thin walls react markedly to speed variations, while for thicker walls this dependence is noticeably weaker.

**Figure 8:**
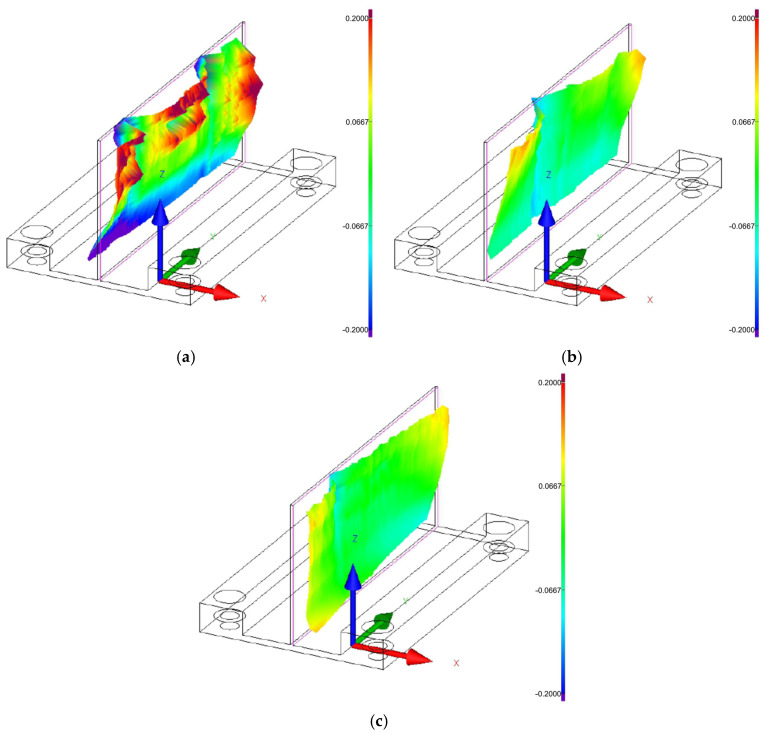
Three-dimensional graphical representation of flatness deviation for a cutting speed of *v_c_* = 300 m/min: (**a**) wall thickness *t* = 1 mm; (**b**) wall thickness *t* = 1.5 mm; (**c**) wall thickness *t* = 2 mm.

**Figure 9:**
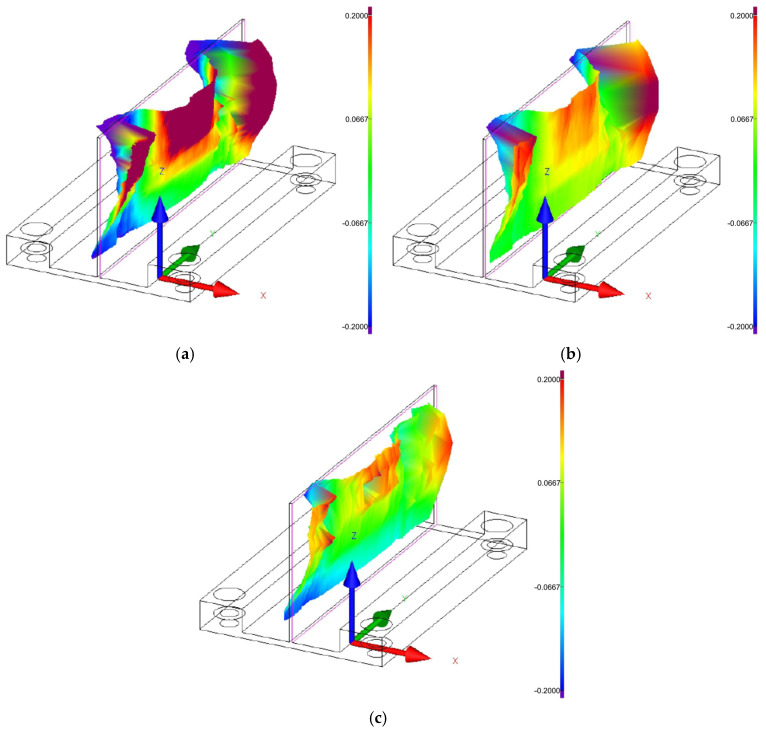
Three-dimensional graphical representation of flatness deviation for a cutting speed of *v_c_* = 600 m/min: (**a**) wall thickness *t* = 1 mm; (**b**) wall thickness *t* = 1.5 mm; (**c**) wall thickness *t* = 2 mm.

**Figure 10:**
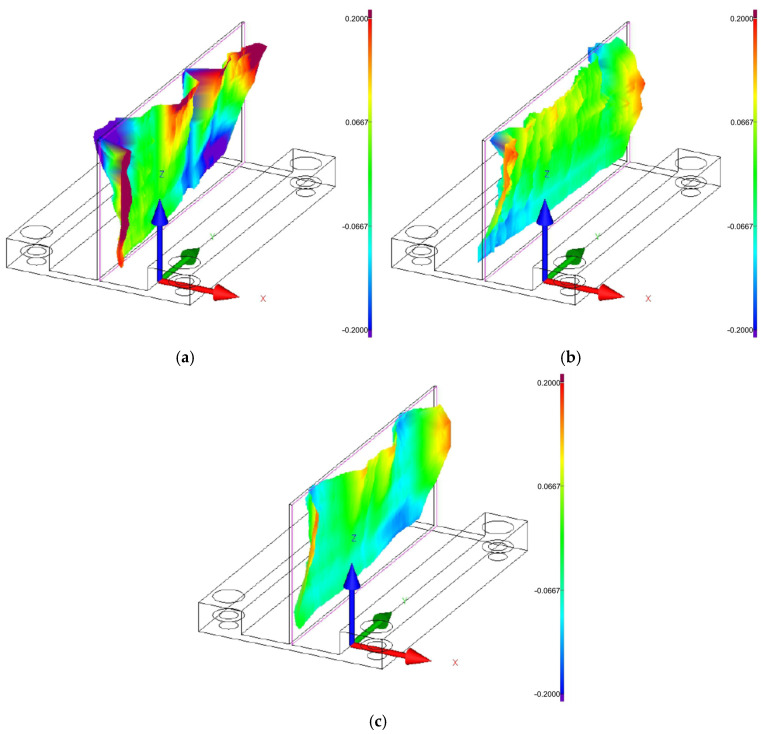
Three-dimensional graphical representation of flatness deviation for a cutting speed of *v_c_* = 900 m/min: (**a**) wall thickness *t* = 1 mm; (**b**) wall thickness *t* = 1.5 mm; (**c**) wall thickness *t* = 2 mm.

Figure 8, Figure 9 and Figure 10 show the 3D graphical representation of flatness deviation for the analysed cutting speeds (*v_c_* = 300 m/min, *v_c_* = 600 m/min, and *v_c_* = 900 m/min) and wall thicknesses (*t* = 1 mm, *t* = 1.5 mm, and *t* = 2 mm). The images present the post-machining deformation of the wall relative to the ideal reference plane. The results demonstrate that irrespective of the applied wall thickness and cutting speed, the thin wall undergoes distortion particularly in the upper part, particularly on its ends, which indicates the most sensitive area of the wall. It can be observed that the lowest flatness deviation was obtained for the highest tested wall thickness, *t* = 2 mm, and for the lowest analyzed cutting speed, *v_c_* = 300 m/min. Also, one can observe the presence of characteristic vertical marks (perpendicular to the tool feed direction) on the wall surface, which probably results from chatter. For the cutting speed *v_c_* = 300 m/min, one such mark can be observed for wall thicknesses *t* = 1.5 mm and *t* = 2 mm, while two marks are observed at cutting speeds *v_c_* = 300 m/min for wall thickness *t* = 1 mm, *v_c_* = 600 m/min, and *v_c_* = 900 m/min.

The results of flatness deviation taken as a post-machining deformation indicator made it possible to establish that the two independent variables have an impact on the thin wall flatness deviation.

### 3.2. Wall Thickness Deviation

The subsequent stage of the analysis concentrated on wall thickness deviation, which—similarly to flatness deviation—was used as an indicator of the post-machining deformation of a thin wall. First of all, the experimental results were processed, and an empirical equation was established for predicting this dependent variable in relation to the tested independent variables. To that end, ANOVA was performed, showing the quadratic model to have the best fit, yielding the coefficients *R*^2^ = 0.9936, adjusted *R*^2^ = 0.9891, and predicted *R*^2^ = 0.9626. In addition, the adequate precision value of 51.1711 confirms the high signal quality level. Ultimately, the empirical regression model describing the wall thickness deviation (Δ*t*) as a function of wall thickness and cutting speed was expressed by the following Equation with coded variables (6):

(6) $$\Delta t=0.3926-0.1437A+0.0222B+0.0045AB+0.0117A^{2}-0.1218B^{2}$$

The developed response surface quadratic model was verified by ANOVA. The obtained *F*-value of 218.61 confirmed the model’s statistical significance. Moreover, there is also only a 0.01% probability that such a high value could result from external noise, rather than due to the actual relationships. An analysis of the *p*-value showed that most model terms were statistically significant (*p* < 0.05). It was established that the desired wall thickness was a key factor affecting wall thickness deviation. The effect of cutting speed was significant, too, yet to a lesser extent. For this case, no statistical significance was confirmed for the *AB* interaction. In addition, the model term *A*^2^ did not reach the level of significance either, which indicates that the relationship between the wall thickness and this deviation is predominantly linear. Given the lack of fit *F*-value of 1.39, it was determined that the lack of fit was not statistically significant relative to the pure error, which means that the model shows a good fit to the experimental data. Table 7 lists a detailed ANOVA results for the response surface quadratic model of wall thickness deviation.

**Figure 11:**
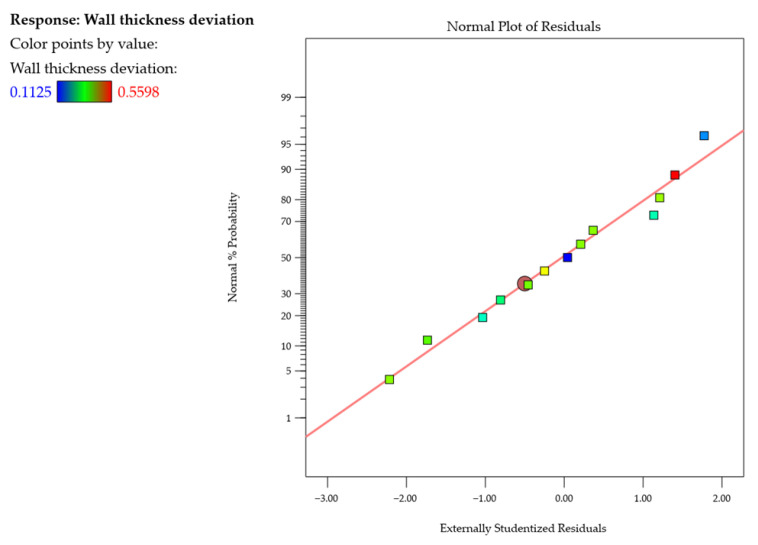
Normal probability plot of studentized residuals for the wall thickness deviation.

After that, the normal probability plot of studentized residuals (Figure 11) was examined, showing no deviations from the assumed normal distribution and finding the model to be adequate.

**Figure 12:**
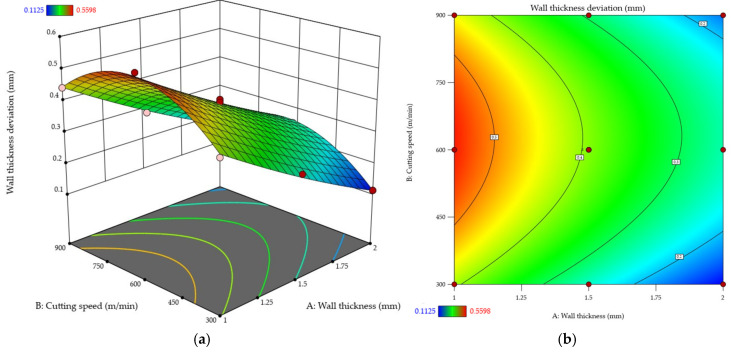
Wall thickness deviation as a function of wall thickness and cutting speed: (**a**) response surface plot; (**b**) contour plot.

The obtained results were plotted (Figure 12) using response surface (Figure 12a) and contour (Figure 12b) plots. An analysis reveals that an increase in the wall thickness reduces the wall thickness deviation. The impact of cutting speed was the same as that observed for flatness deviation. For each tested thickness, the use of a higher cutting speed leads to a greater wall thickness deviation, with the deviation value being the highest at about *v_c_* ≈ 600 m/min and then decreasing. It must be emphasized that at the highest analyzed cutting speed, *v_c_* = 900 m/min, the wall thickness deviation is still higher than that obtained at the lowest cutting speed, *v_c_* = 300 m/min. This behaviour pattern is particularly evident for the wall thickness *t* = 1 mm, where the cutting speed increase contributes to a significant rise in the deviation value. With thicker walls, this effect is less pronounced, and the differences between the values acquired for thicknesses *t* = 1.5 mm and *t* = 2 mm are considerably smaller compared to *t* = 1 mm.

The results of wall thickness deviation, another indicator of the post-machining deformation of a thin wall, showed that both independent variables affected the value of this indicator.

### 3.3. Residual Stress

The final stage of the analysis focused on residual stress, which is an important aspect of studies on the post-machining deformation of thin-walled components. The dependent variable was measured in two directions: *x* and *y*, denoted by $\sigma_{x}$ (*x*-axis) and $\sigma_{y}$ (*y*-axis), respectively. This allowed for an in-depth assessment of the surface stress state. As previously mentioned, the first step was a statistical analysis of the experimental data and finding empirical equations for predicting the residual stress as a response to wall thickness and cutting speed. ANOVA was used to that end, showing the quadratic model to yield the best fit for both directions: *x* $\sigma_{x}$ and *y* $\sigma_{y}$. For the residual stress $\sigma_{x}$, the coefficients were as follows: *R*^2^ = 0.9631, adjusted *R*^2^ = 0.9368 and predicted *R*^2^ = 0.7425, whereas for the residual stress $\sigma_{y}$ the coefficients were: *R*^2^ = 0.9620, adjusted *R*^2^ = 0.9349 and predicted *R*^2^ = 0.7635. The slightly lower predicted *R*^2^ can be attributed to the high sensitivity of residual stress to small variations in machining conditions and the inherent variability of XRD measurements. The adequate precision values were 20.7350 and 17.2547, which confirms the adequate signal level. The empirical regression models for the residual stresses $\sigma_{x}$ and $\sigma_{y}$ were described by Equations (7) and (8) using coded variables:

(7) $$\sigma_{x}=49.62-40.17A+15.67B-11.25AB+41.33A^{2}-56.17B^{2}$$

(8) $$\sigma_{y}=186.10-43.33A+29.17B-15.25AB-8.86A^{2}-99.36B^{2}$$

The assumed response surface quadratic models for residual stresses $\sigma_{x}$ and $\sigma_{y}$ were verified via ANOVA. The model *F*-value of 36.56 for $\sigma_{x}$ and 35.48 for $\sigma_{y}$ indicate a high statistical significance of the adopted models, and the probability of the results being distorted by noise is only 0.01%. An additional analysis of the *p*-value showed most terms in both models to be statistically significant (*p* < 0.05). Thin wall thickness bears the most significance for the residual stress along both *x* $\sigma_{x}$ and *y* $\sigma_{y}$. The cutting speed also affects the results, albeit to a less significant extent. Regarding the residual stress $\sigma_{x}$, the exception is the *AB* interaction, which exhibits no statistical significance. For the residual stress $\sigma_{y}$, apart from the above-mentioned *AB* interaction, *A*^2^ holds no statistical significance either. An analysis of the lack of fit for the *F*-values of 3.14 and 1.78 showed that they were not statistically significant, which confirms that the numerical models reproduced the experimental data accurately. Detailed ANOVA results for the response surface quadratic model regarding the residual stresses $\sigma_{x}$ and $\sigma_{y}$ are listed in Table 8 and Table 9.

**Figure 13:**
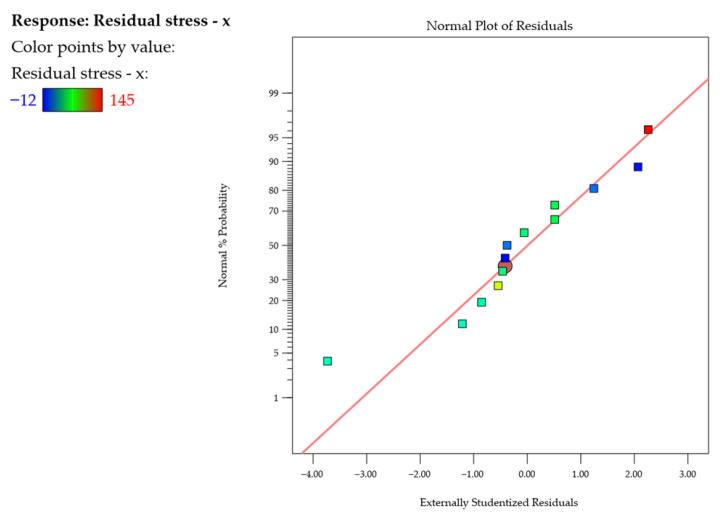
Normal probability plot of studentized residuals for the residual stress $\sigma_{x}$.

**Figure 14:**
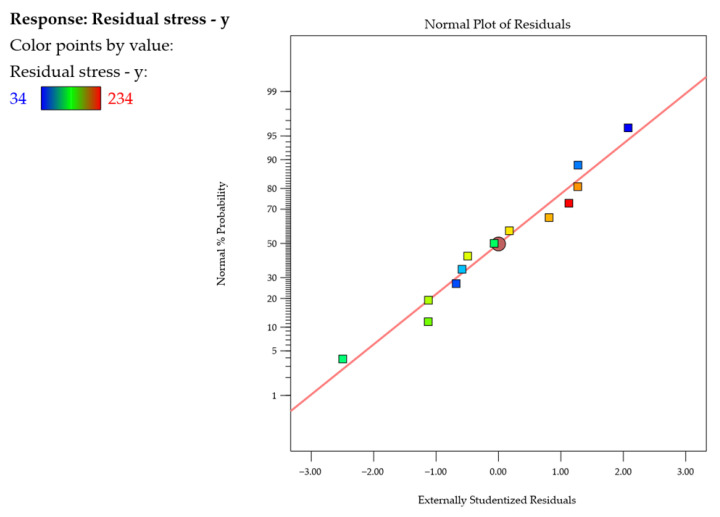
Normal probability plot of studentized residuals for the residual stress $\sigma_{y}$.

Next, the normal probability plots of studentized residuals (Figure 13 and Figure 14) were examined, showing neither response transformations nor evident problems with normal distributions. Also, the adopted models were found to be adequate.

**Figure 15:**
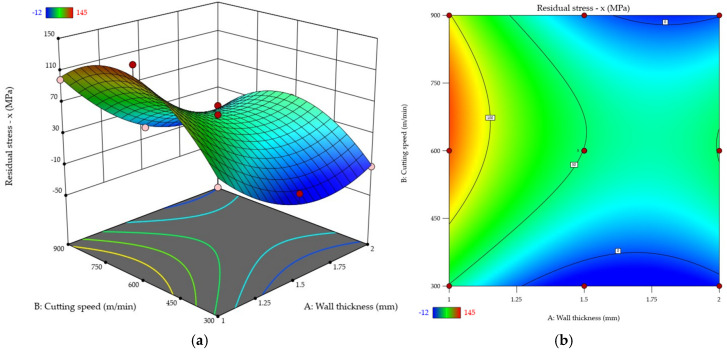
Residual stress $\sigma_{x}$ as a function of wall thickness and cutting speed: (**a**) response surface plot; (**b**) contour plot.

**Figure 16:**
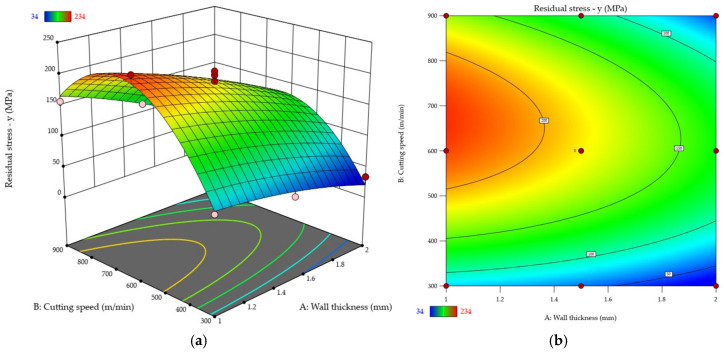
Residual stress $\sigma_{y}$ as a function of wall thickness and cutting speed: (**a**) response surface plot; (**b**) contour plot.

For clarity, the results of the residual stresses $\sigma_{x}$ and $\sigma_{y}$ were plotted (Figure 15 and Figure 16). Response surface (Figure 15a and Figure 16a) and contour (Figure 15b and Figure 16b) plots were used to that end. The diagrams revealed that the residual stress values were higher in the direction *y* $\sigma_{y}$ rather than along *x* $\sigma_{x}$. Additionally, for most cases, the tensile residual stress was predominant. The compressive residual stress was only noted for the cutting speed *v_c_* = 300 m/min for two wall thicknesses: *t* = 1.5 mm and *t* = 2 mm (in *x* direction). An analysis of the results also showed that the residual stresses $\sigma_{x}$ and $\sigma_{y}$ decrease with increasing wall thickness. Whatever the thin wall thickness value, the use of a higher cutting speed results in elevated residual stresses $\sigma_{x}$ and $\sigma_{y}$. The maximum residual stress value occurs between the cutting speeds *v_c_* = 600 m/min and *v_c_* = 750 m/min; after that, the stress decreases, yet not that dramatically. The most characteristic behaviour pattern was observed for the wall thickness *t* = 1 mm.

The results of the residual stresses $\sigma_{x}$ and $\sigma_{y}$ confirm that the two tested independent variables have an impact on the dependent variable, which agrees with previous findings.

## 4. Discussion

The findings of this study reveal that both wall thickness and cutting speed have a significant impact on flatness and wall thickness deviations (post-machining deformation indicators), as well as on the residual stress on the surface of a thin wall made of aluminum alloy 7050 T7451.

The flatness and wall thickness deviations results clearly show that the thin wall with a thickness of *t* = 1 mm is particularly susceptible to post-machining deformation, which agrees with the observations reported in the literature regarding the low stiffness of such components [8]. An increase in the wall thickness to *t* = 1.5 mm, and especially to *t* = 2 mm, caused a noticeable reduction in this effect. This suggests the presence of a minimal wall thickness that allows for performing milling at a maximum axial depth of cut without any serious risk of exceeding the allowable dimensional and geometrical tolerances. In addition to that, for the thin wall one can probably observe the presence of chatter-induced marks that considerably reduce the shape and dimensional accuracy of finished parts and their surface quality [65,66]. The wall height of 48 mm is also an important aspect. It should be stressed that there are limited studies strictly devoted to thin wall milling with a maximum axial depth of cut.

The results of cutting speed analysis demonstrate that the highest post-machining deformation of the thin wall occurred at approximately *v_c_* ≈ 600 m/min, which can be attributed to the influence of the cutting force. The observed reduction in the post-machining deflection of the thin wall after this cutting speed value is probably a result of entering the high-speed cutting range, which leads to a lower cutting force [50]. It should be noted that this transition from conventional machining to high-speed cutting is a characteristic feature of a given material. Importantly, the post-machining deformation of the thin wall formed at the cutting speed *v_c_* = 900 m/min was still higher than that at *v_c_* = 300 m/min, which may result from both enhanced thermal effects [31], and also probably from the appearance of chatter [67]. This indicates that the use of high-speed cutting for such part geometry does not prevent the problem but merely reduces its scale.

The results of residual stress measurements in two directions, $\sigma_{x}$ and $\sigma_{y}$, serve for drawing inferences about the mechanisms governing the post-machining deformation of thin-walled components. The residual stress is higher in the direction *y* $\sigma_{y}$ rather than along *x* $\sigma_{x}$, and in both cases, tensile stress is predominant. Whatever the wall thickness, the stress values were the highest at the cutting speed *v_c_* ≈ 600 m/min. This is presumably a result of the strong impact of thermal and mechanical interactions, particularly heat in the cutting zone, which generates primarily tensile stress [68,69]. The occurrence of compressive stress (in *x* direction) observed at *v_c_* = 300 m/min for the wall thicknesses *t* = 1.5 mm and *t* = 2 mm can be ascribed to the prevailing influence of the mechanical impact of the cutting edge over thermal effects [24]. It should be noted that Jiang et al. [70] stressed that cutting force was responsible for inducing both compressive and tensile residual stress, whereas the thermal influence of milling primarily promoted the development of tensile residual stress. One must also draw attention to the effect of thin wall thickness on residual stress, i.e., the residual stresses (both $\sigma_{x}$ and $\sigma_{y}$) decreased as the wall thickness was increased.

It must be highlighted that the observed changes in the residual stress and post-machining deformation of thin-walled components reflect the complex nature of the milling process. This is confirmed by the applied response surface quadratic models, which showed a good fit to the experimental data (for most cases, the determination coefficient *R*^2^ > 0.96). The results suggest that in industrial practice, the machining parameters for thin-walled structures should be optimized taking into account both cutting speed and wall thickness, so that milling could be carried out at a maximum axial depth of cut.

## 5. Conclusions

Based on the results of this study, the following conclusions can be drawn:

- Although thin wall milling at a maximum axial depth of cut is feasible, it largely depends on wall thickness. For the wall thickness *t* = 1 mm, one could observe the formation of considerable post-machining deformation, while the wall thickness *t* ≥ 1.5 mm, with the height of 48 mm, made it possible to achieve the required shape and dimensional accuracy.
- Cutting speed has a significant impact on the post-machining deformation of a thin wall and residual stress. For each tested wall thickness, the dependent variables reached the maximum values when the cutting speed was about *v_c_* ≈ 600 m/min, followed by their decrease. For the cutting speed *v_c_* = 900 m/min, the post-machining deformation of the wall reduced (compared to *v_c_* = 600 m/min); nevertheless, it was still higher than the distortion obtained at *v_c_* = 300 m/min.
- The examination of the thin-walled part revealed the predominance of tensile stress on the surface, its values being higher in the direction *y* $\sigma_{y}$ rather than along *x* $\sigma_{x}$. The highest residual stress occurred at *v_c_* ≈ 600 m/min, which probably results from thermal interactions, whereas at *v_c_* = 300 m/min and the wall thickness *t* ≥ 1.5 mm, the compressive stress prevailed (in *x* direction), which was connected with the dominant impact of the cutting edge. In contrast, an increase in the wall thickness caused a decrease in the residual stress.
- The response surface regression models showed a very good fit to the experimental results (*R*^2^ > 0.96), which confirms that they can be used for predicting the post-machining deformation of thin walls and residual stress under similar machining conditions.
- The results of this study have a practical application in industry, particularly the aerospace branch, demonstrating that the optimization of milling performed at a maximum axial depth of cut should include selecting the appropriate cutting speed and wall thickness already in the part design stage. The results can serve as a basis for developing technological guidelines for machining parts made of aluminum alloys.

Future studies should focus on analyzing the impact of thermal and mechanical interactions occurring during thin wall milling at a maximized axial depth of cut, with a particular emphasis on their effect on the formation of residual stress and post-machining deformation. Furthermore, it would be advisable to investigate the process dynamics.

## Figures and Tables

**Table 1:** Compilation of relevant studies on minimizing the post-machining deformation of thin-walled elements.

Material	Machining Parameters	Method of Minimizing Post-Machining Deformation	Reference
Aluminum alloy EN AC-46000	Numerical machining parameters: *a_p_* = 8.5 and 17 mm *a_e_* = 0.2–1.4 mm *v_f_* = 80–320 mm/min *f_z_* = 0.05–0.2 mm/tooth Numerical tool parameters: tool 1: *d*_1_ = 12 mm, *z* = 2 tool 2: *d*_1_ = 20 mm, *z* = 2 Experimental machining parameters: tool 1: *a_e_* = 1.4 and 1 mm, *a_p_* = 17 mm, *f_z_* = 0.2 and 0.05 mm/tooth, *v_f_* = 320 and 80 mm/min tool 2: *a_e_* = 0.6 mm, *a_p_* = 17 mm, *f_z_* = 0.05 mm/tooth, *v_f_* = 80 mm/min Experimental tool parameters: tool 1: *d*_1_ = 12 mm, *z* = 2 tool 2: *d*_1_ = 20 mm, *z* = 2	Suitable machining strategy selection; in this study, the waterline strategy (machining is performed on one side of the wall first and then on the other) yields better machining results than the side-by-side strategy (machining is performed on both sides of the wall, once on one side and once on the other). It is also recommended to use a cutting tool with a bigger diameter.	[39]
Aluminum alloy A5052 (denoted according to JIS)	Dry up-cut machining parameters: *a_e_* = 0.2 mm *a_p_* = 15 mm *n* = 2275 rpm *f_z_* = 0.055 mm/tooth Tool parameters (end mill): *d* = 14 mm *z* = 3 *κ_r_* = 40°	Cutting tool path optimization and material removal in a strictly defined sequence of machining passes via the application of an appropriate machining sequence for material blocks along the wall.	[40]
Aluminum alloy 5083	Machining parameters: *n* = 1500 rpm *v_f_* = 200 mm/min *a_p_* = 2 mm Tool parameters (solid carbide mill): *d* = 4 mm *z* = 4 *κ_r_* = 30°	Use of a parallel spiral tool path to produce the best machining effects in terms of manufacturing quality of a thin-walled element.	[41]
Aluminum alloy 6061-T6	Machining parameters: *n* = 2000 rpm *v_f_* = 300 mm/min *a_e_* = 4 mm *a_p_* = 4–25 mm Tool parameters (solid carbide mill): *d* = 16 mm *z* = 4 *κ_r_* = 30°	Machining parameters optimization, i.e., axial depth of cut and radial depth of cut (milling width).	[42]
Aluminum alloy 6061	Machining parameters: *n* = 8000 rpm *v_f_* = 1000 mm/min *a_p_* = 0.5 mm Tool parameters: *d* = 20 mm *z* = 2	Use of mirror milling, wherein the opposite side of the machined wall is supported. A system for online measurement of wall thickness is devised.	[43]
Aluminum alloy 7075	Machining parameters: *v_c_* = 200 m/min *v_f_* = 200 mm/min Experimental tool parameters (carbide ball nose end mill): *d* = 8 mm *z* = 4 *κ_r_* = 30° Wall thickness: *g* = 2.15 mm	Tool path compensation in a virtual environment after the use of the proposed simulation methodology to predict the post-machining deformation of thin-walled elements.	[44]
Aluminum alloy 7075 and titanium alloy Ti6Al4V	9 standard thin-walled structures with different geometries were analyzed, and the machining effects of their manufacture were determined for the following variants: variant 1: machining of a semi-finished product and direct achievement of the final shape variant 2: machining of a semi-finished product and leaving the stiffening ribs, and then performing 96 h-long natural ageing at room temperature, followed by another machining operation to remove the stiffening ribs variant 3: machining of a semi-finished product and leaving the stiffening ribs, and then performing a combination of heat treatment and vibratory treatment for residual stress relaxation, followed by another machining operation to remove the stiffening ribs	Use of a suitable technological process for milling a thin-walled component with stiffening ribs in the first operation and then leaving it for 96 h, or performing a combination of heat treatment and vibratory treatment for residual stress relaxation, followed by another milling operation to remove the stiffening ribs. In addition, topological optimization of the arrangement of stiffening ribs is proposed.	[45]
Titanium alloy Ti6Al4V	Machining parameters: *n* = 1300 rpm *v_f_* = 416 mm/min *a_p_* = 15 mm Tool parameters (solid carbide mill): *d* = 20 mm *z* = 4 *κ_r_* = 40°	Airflow applied on the opposite side of the wall relative to the machined surface for additional reinforcement.	[46]
Titanium alloy Ti6Al4V	Machining parameters: *n* = 4000–10,000 rpm *f* = 0.1–0.25 mm/rev *a_p_* = 1–4 mm *a_e_* = 1–6 mm Tool parameters (straight shank end mill with cemented carbide material): *d* = 20 mm *z* = 3 *κ_r_* = 45°	Machining parameters optimization, including rotational speed, axial depth of cut, radial depth of cut (milling width), and feed rate.	[47]
Titanium alloy Ti6Al4V	Machining parameters: roughing: *n* = 800 rpm, *f_z_* = 0.3 mm/tooth, *a_p_* = 5 mm finishing: *n* = 1200 rpm, *f_z_* = 0.2 mm/tooth, *a_p_* = 0.2 mm Tool parameters (hard-alloy flat-end cutter): *d* = 16 mm *z* = 4 *κ_r_* = 45°	Special clamping solution for machining thin-walled parts is proposed, with critical components of the clamp made of polyetheretherketone (PEEK-GF30).	[48]
Titanium alloy (unspecified)	Numerical down-cut machining parameters: *a_p_* = 1 mm *n* = 1500 rpm *v_f_* = 1000–3000 mm/min Numerical tool parameters: *d* = 10 mm Experimental down-cut machining parameters: roughing: *n* = 1500 rpm, *v_f_* = 3000 mm/min finishing: *n* = 1500 rpm, *v_f_* = 1000 mm/min Experimental tool parameters (end mill): roughing: *d* = 15 mm and *z* = 4 finishing: *d* = 10 mm and *z* = 4	Tool path optimization and the use of a ring-cutting tool path for both cutting force and machining time reduction, leading to higher productivity. It is suggested that the workpiece be removed from the clamp after roughing to release residual stress and then reclamped for finishing.	[49]

**Table 2:** Independent variables and their coded levels for the face-centred central composite design (FCCCD).

Independent Variables	Codes	Levels of Coded Variables		
		Low	Medium	High
		−1	0	+1
Wall thickness *t* [mm]	*A*	1	1.5	2
Cutting speed *v_c_* [m/min]	*B*	300	600	900

**Table 3:** Chemical composition of 7050 T7451 aluminum alloy (compiled based on [64]).

Chemical Composition [%]											
Si	Fe	Cu	Mn	Mg	Cr	Zn	Ti	Zr	Others	Others Total	Al
0.05	0.07	2.20	0.01	2.10	0.01	6.30	0.03	0.10	0.01	0.03	The rest

**Table 4:** Technological parameters of finishing.

Cutting Parameters	Roughing	Finishing
Cutting speed *v_c_* [m/min]	900	300, 600, 900
Feed per tooth *f_z_* [mm/tooth]	0.05	0.025
Axial depth of cut *a_p_* [mm]	5 and 3 (for the final tool pass)	48
Radial depth of cut (milling width) *a_e_* [mm]	8.9 and 10.4 (for a wall thickness of 1 mm)	0.2
	8.65 and 10.4 (for a wall thickness of 1.5 mm)	
	8.4 and 10.4 (for a wall thickness of 2 mm)	

**Table 5:** Detailed technical parameters of cutting tools.

Technical Parameters	44985	44748
Cutting diameter [mm]	16	12
Shank diameter [mm]	16	12
Length of cut [mm]	35	48
Overall length [mm]	108	100
Helix angle [°]	Variable	41
Number of flutes [-]	3	4
Coating	TiB_2_	TiB_2_

**Table 6:** ANOVA for the response surface quadratic model of flatness deviation.

Source	Sum of Squares	df	Mean Square	*F*-Value	*p*-Value	
**Model**	0.1011	5	0.0202	112.15	<0.0001	significant
*A*-Wall thickness	0.0186	1	0.0186	103.42	<0.0001	
*B*-Cutting speed	0.0053	1	0.0053	29.63	0.0010	
*AB*	0.0000	1	0.0000	0.1618	0.6995	
*A* ^2^	0.0019	1	0.0019	10.39	0.0146	
*B* ^2^	0.0729	1	0.0729	404.50	<0.0001	
**Residual**	0.0013	7	0.0002			
Lack of Fit	0.0010	3	0.0003	4.90	0.0793	not significant
Pure Error	0.0003	4	0.0001			
**Cor Total**	0.1023	12				

Standard deviation: 0.0134, mean: 0.3454, coefficient of variation %: 3.89, *R*^2^: 0.9877, adjusted *R*^2^: 0.9789, predicted *R*^2^: 0.8976, adequate precision: 33.6032.

**Table 7:** ANOVA for the response surface quadratic model of wall thickness deviation.

Source	Sum of Squares	df	Mean Square	*F*-Value	*p*-Value	
**Model**	0.1718	5	0.0344	218.61	<0.0001	significant
*A*-Wall thickness	0.1239	1	0.1239	788.46	<0.0001	
*B*-Cutting speed	0.0030	1	0.0030	18.82	0.0034	
*AB*	0.0001	1	0.0001	0.5098	0.4984	
*A* ^2^	0.0004	1	0.0004	2.42	0.1635	
*B* ^2^	0.0409	1	0.0409	260.57	<0.0001	
**Residual**	0.0011	7	0.0002			
Lack of Fit	0.0006	3	0.0002	1.39	0.3674	not significant
Pure Error	0.0005	4	0.0001			
**Cor Total**	0.1729	12				

Standard deviation: 0.0125, mean: 0.3419, coefficient of variation %: 3.67, *R*^2^: 0.9936, adjusted *R*^2^: 0.9891, predicted *R*^2^: 0.9626, adequate precision: 51.1711.

**Table 8:** ANOVA for the response surface quadratic model of the residual stress $\sigma_{x}$.

Source	Sum of Squares	df	Mean Square	*F*-Value	*p*-Value	
**Model**	21,656.93	5	4331.39	36.56	<0.0001	significant
*A*-Wall thickness	9680.17	1	9680.17	81.70	<0.0001	
*B*-Cutting speed	1472.67	1	1472.67	12.43	0.0097	
*AB*	506.25	1	506.25	4.27	0.0776	
*A* ^2^	4717.25	1	4717.25	39.81	0.0004	
*B* ^2^	8714.75	1	8714.75	73.55	<0.0001	
**Residual**	829.38	7	118.48			
Lack of Fit	582.18	3	194.06	3.14	0.1490	not significant
Pure Error	247.20	4	61.80			
**Cor Total**	22,486.31	12				

Standard deviation: 10.88, mean: 42.77, coefficient of variation %: 25.45, *R*^2^: 0.9631, adjusted *R*^2^: 0.9368, predicted *R*^2^: 0.7425, adequate precision: 20.7350.

**Table 9:** ANOVA for the response surface quadratic model for the residual stress $\sigma_{y}$.

Source	Sum of Squares	df	Mean Square	*F*-Value	*p*-Value	
**Model**	51,619.14	5	10,323.83	35.48	<0.0001	significant
*A*-Wall thickness	11,266.67	1	11,266.67	38.73	0.0004	
*B*-Cutting speed	5104.17	1	5104.17	17.54	0.0041	
*AB*	930.25	1	930.25	3.20	0.1169	
*A* ^2^	216.91	1	216.91	0.7456	0.4165	
*B* ^2^	27,267.79	1	27,267.79	93.72	<0.0001	
**Residual**	2036.55	7	290.94			
Lack of Fit	1164.55	3	388.18	1.78	0.2899	not significant
Pure Error	872.00	4	218.00			
**Cor Total**	53,655.69	12				

Standard deviation: 17.06, mean: 136.15, coefficient of variation %: 12.53, *R*^2^: 0.9620, adjusted *R*^2^: 0.9349, predicted *R*^2^: 0.7635, adequate precision: 17.2547.

## Data Availability

The original contributions presented in this study are included in the article. Further inquiries can be directed to the corresponding author.

## Appendix A

**Table A1:** Experimental design and results of face-centred central composite design (FCCCD).

Run	Actual Variable 1: Wall Thickness *A* [mm]	Coded Variable 1: Wall Thickness *A* [-]	Actual Variable 2: Cutting Speed *B* [m/min]	Coded Variable 2: Cutting Speed *B* [-]	Response 1: Flatness Deviation *f_d_* [mm]	Response 2: Wall Deviation Δ*t* [mm]	Response 3: Residual Stress—*x* *σ_x_* [MPa]	Response 4: Residual Stress—*y* *σ_y_* [MPa]
1	1	−1	300	−1	0.2912	0.3984	37	72
2	1.5	0	300	−1	0.2116	0.2586	−9	49
3	1.5	0	600	0	0.4208	0.3752	55	199
4	1	−1	900	+1	0.3470	0.4425	99	157
5	2	+1	600	0	0.3596	0.2515	47	111
6	1.5	0	600	0	0.4096	0.3952	55	189
7	1.5	0	900	+1	0.2732	0.2857	6	115
8	1	−1	600	0	0.5072	0.5598	145	234
9	2	+1	300	−1	0.1924	0.1125	−12	34
10	1.5	0	600	0	0.4109	0.4060	41	205
11	2	+1	900	+1	0.2590	0.1745	5	58
12	1.5	0	600	0	0.3998	0.3871	49	178
13	1.5	0	600	0	0.4025	0.3971	38	169
